# Vector-Virus Interactions and Transmission Dynamics of West Nile Virus

**DOI:** 10.3390/v5123021

**Published:** 2013-12-09

**Authors:** Alexander T. Ciota, Laura D. Kramer

**Affiliations:** 1 The Arbovirus Laboratories, Wadsworth Center, New York State Department of Health, Slingerlands, NY 12159, USA; E-Mail: aciota@wadsworth.org; 2 Department of Biomedical Sciences, School of Public Health, SUNY, Albany, NY 12201, USA

**Keywords:** *West Nile virus*, vectorial capacity, Culex, host competence, mosquito biology, virus evolution

## Abstract

*West Nile virus* (WNV; *Flavivirus*; *Flaviviridae*) is the cause of the most widespread arthropod-borne viral disease in the world and the largest outbreak of neuroinvasive disease ever observed. Mosquito-borne outbreaks are influenced by intrinsic (e.g., vector and viral genetics, vector and host competence, vector life-history traits) and extrinsic (e.g., temperature, rainfall, human land use) factors that affect virus activity and mosquito biology in complex ways. The concept of vectorial capacity integrates these factors to address interactions of the virus with the arthropod host, leading to a clearer understanding of their complex interrelationships, how they affect transmission of vector-borne disease, and how they impact human health. Vertebrate factors including host competence, population dynamics, and immune status also affect transmission dynamics. The complexity of these interactions are further exacerbated by the fact that not only can divergent hosts differentially alter the virus, but the virus also can affect both vertebrate and invertebrate hosts in ways that significantly alter patterns of virus transmission. This chapter concentrates on selected components of the virus-vector-vertebrate interrelationship, focusing specifically on how interactions between vector, virus, and environment shape the patterns and intensity of WNV transmission.

## 1. Introduction

*West Nile virus* (WNV; *Flaviviridae*; *Flavivirus*) was first detected in the Americas in 1999, the result of a single point introduction into the New York City area [1,2], followed by a dramatic range expansion [3,4,5,6]. This virus is currently the most widely distributed arbovirus in the world, occurring on all continents except Antarctica. Throughout its worldwide distribution, WNV is maintained in nature in an enzootic cycle between competent ornithophilic mosquito vectors—predominantly Culex species and susceptible birds—the particular species dependent on geographic location. In all, 59 species of mosquitoes and 284 species of birds have been found infected in North America [4]. Virus prevalence varies spatially and temporally depending on a complex set of intrinsic and extrinsic factors. Intrinsic factors which are influenced by interspecies and intraspecies genetics include such elements as host and vector competence, mosquito feeding preferences, mosquito longevity, and host immunity [7] as has been thoroughly reviewed by [8]. Extrinsic factors influence contact between the mosquito and susceptible vertebrate hosts and include such elements as density and composition of the host and vector populations, and environmental conditions. These factors together contribute to selective pressures shaping dynamic viral populations, host-virus outcomes and, ultimately, epidemiological patterns.

WNV has been successful in persisting in the environment of North America, partly a consequence of its generalist nature, but also likely a consequence of its ability to adapt readily to new hosts. Like all RNA viruses, WNV has the ability to mutate rapidly due to its error-prone RNA dependent RNA polymerase, rapid rate of replication, and replication to high titers. This chapter will delve into intrinsic and extrinsic factors, focusing on vector-virus-environment interactions that impact the intensity of WNV transmission. In evaluating the importance of each factor, it is critical to do so in the context of vectorial capacity (VC), a measure of a mosquito population’s capacity to transmit an infectious agent to a new susceptible population. Calculation of VC is founded on the basic formula:

VC = ma^2^bp^n^/-log_e_ p (1)

where m = number of female mosquitoes per host, a = daily blood feeding rate, b = transmission rate among exposed mosquitoes, p = the probability of daily survival, n = extrinsic incubation period, based on [9]. A number of modifications that acknowledge geographical, ecological, and epidemiological complexities can be added to improve accuracy of the estimate (see [10,11]). Here, we review current knowledge of the dynamic and complex nature of factors influencing WNV vectorial capacity, as well as how these factors broadly impact vertebrate host infections and competence. 

## 2. Vector

### 2.1. Vector Distribution and Genetics

Recently available molecular tools have facilitated advancement of our knowledge of medically important mosquito vector genetics, feeding habits, and infection prevalence. Species distribution and population density suggest that *Culex pipiens* L. and *Culex restuans* Theobald may be responsible for up to 80% of human WNV infections in the northeastern USA [12]. *Cx. restuans* populations peak in the spring and early summer [13,14,15], unlike *Cx. pipiens* populations which peak later, and the former may be the critical vector of WNV early in the transmission season [16]. *Cx. restuans* occurs from California to North Carolina, from southern Canada to Honduras; locally adapted populations may differ in epidemiologically significant traits [17]. 

**Figure 1:**
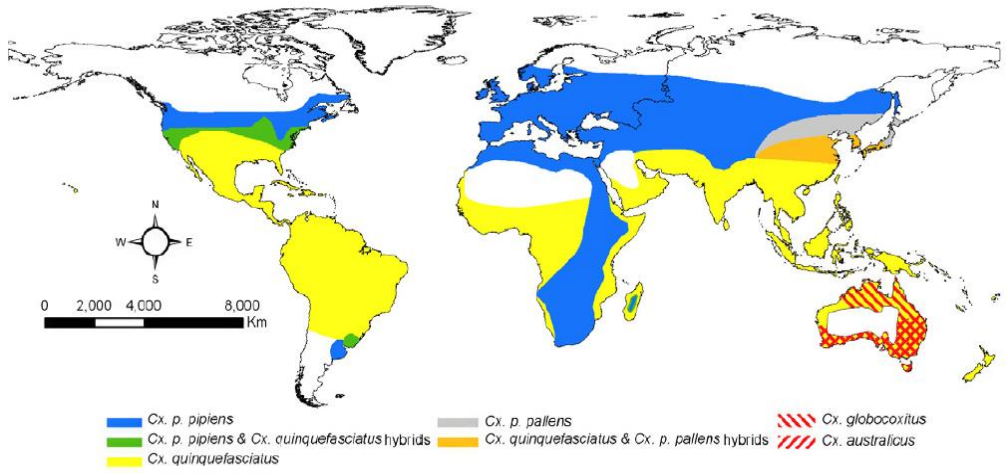
Global distribution of *Cx. pipiens* complex mosquitoes. Geographic range for *Cx. p. pipiens* includes both forms (pipiens and molestus). *Cx. australicus* and *Cx. globocoxitus* are restricted to Australia. Taken from [22].

The *Cx. pipiens* complex includes *Cx. pipiens*, *Cx. quinquefasciatus* Say, *Cx. australicus* Dobrotworsky & Drummond, and *Cx. globocoxitus* Dobrotworsky, mosquito species that have a worldwide distribution (Figure 1), are evolutionarily closely related, and difficult to separate morphologically [18]. There are two recognized subspecies of *Culex pipiens*, *i.e.*, *Cx. pipiens pipiens*, an Old World taxa distributed from Northern Europe to the highlands of South Africa [19] and *Cx. p. pallens*, distributed east of the Urals across temperate Asia [20]. Furthermore, *Cx. pipiens* has two bioforms—molestus and pipiens—which differ in a number of biological characteristics including autogeny, stenogamy, ability to diapause, and feeding preference [21,22]. The sibling species *Cx. quinquefasciatus* differs from *Cx. pipiens* in that it thrives in tropical and sub-tropical regions south of 36° latitude [23,24], but, like *Cx. pipiens*, is predominantly ornithophilic, preferring to feed on birds, although both also will feed on humans. Hybrids of *Cx. pipiens* and *Cx. quinquefasciatus* are found in a zone where the two overlap stretching from approximately 30°N to 40°N latitude in N. America [25,26,27]. Studies of hybrid populations of *Cx. pipiens* complex mosquitoes including *Cx. pipiens* form pipiens, *Cx. pipiens* form molestus, and *Cx. quinquefasciatus* indicate hybridization has a significant effect on WNV infection, dissemination, and, particularly, transmission. Specifically, presence of *Cx. quinquefasciatus* signature in the hybrid mosquitoes increased susceptibility to infection; and the percent of infected hybrid populations transmitting by day 13/14 was found to be significantly higher than one or both parental populations [28]. Therefore, extrinsic factors such as land use and urbanization in particular, which likely increase the potential for hybridization between bioforms [29,30,31], may have an impact on WNV activity. Extensive discussion of the *Cx. pipiens* species complex can be found elsewhere [22,32]. 

The primary WNV vector in the western United States (USA) is *Cx. tarsalis* Coquillett, [23,33]. Unlike *Cx. pipiens*, *Cx. tarsalis* thrive in agricultural landscapes [34] and is responsible for the western expansion of WNV [35], in which human activity was highly correlated to *Cx. tarsalis* activity in rural regions separating urban landscapes [36,37]. *Cx. tarsalis* is not known to hybridize, yet significant spatial genetic clustering is noted [38,39], which likely contributes to population level variability in vectorial capacity.

### 2.2. Vectorial Capacity

#### 2.2.1. Vector Competence (b) and Extrinsic Incubation Period (n)

Following ingestion of an infectious blood meal by a mosquito, the virus must overcome barriers to transmission including the midgut infection barrier [7], midgut escape barrier [40], salivary gland infection barrier [8] and salivary gland escape barrier [41]. Taken together, the ability to overcome these barriers comprises vector competence, and the number of days from ingestion to transmission, the extrinsic incubation period (EIP). Vector competence of a mosquito population can vary over time and appears to be dependent on intrinsic and extrinsic influences, including environmental and genetic factors, as well as their interaction. Although vector competence is not the most important determinant of vectorial capacity, and relatively incompetent populations are capable of sustaining arbovirus outbreaks [42], capacity does fluctuate directly with competence and is therefore represented as a linear term in expressions of vectorial capacity [43,44].

The vector competence of colonized and field populations of mosquitoes for WNV has been a focus of research since WNV was first identified in the USA. It has been found to vary among mosquito species and genera [13,45,46,47,48,49], days since feeding [50,51,52], strain of WNV [50,51,52], and temperature during the EIP [51,53]. Specifically, studies with *Cx. tarsalis* calclulated an EIP of 109 degree-days for WNV [54], yet subsequent work with *Cx. pipiens* demonstrated an accelerating effect with increasing temperatures, suggesting degree day models are likely to underestimate the effects of temperature [51]. Even modest increases in EIP will exponentially increase vectorial capacity independent of intrinsic transmissibility (competence), so temperature, viral genotype, and other factors altering the pace of viral infections in mosquitoes are likely to be of primary importance in governing activity. Vector competence for WNV also has been shown to vary among mosquito populations of the same species [46,55,56,57] with evidence of a genetic basis [58], and may vary seasonally [59]. Spatio-temporal variability in vector competence of *Cx. pipiens* and the possible interaction between mosquito genetics and local environmental factors was demonstrated in NYS [60]. These studies characterize genetic differentiation in *Cx. pipiens* populations between counties in NYS, demonstrating that vector competence is not a static intrinsic trait of a particular mosquito population, but instead a dynamic trait governed by the intersection of multiple variables.

#### 2.2.2. Population Density (m) and Daily Survival (p)

**Figure 2:**
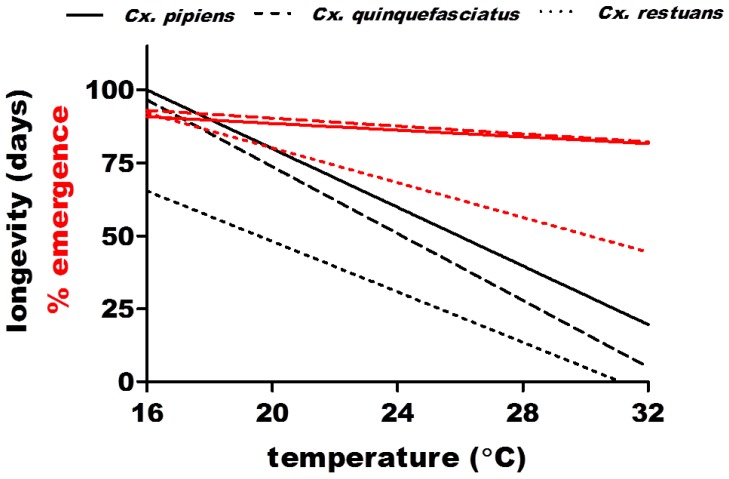
Species-specific differences in adult longevity, immature survival, and effect of temperature in field populations of *Culex pipiens*, *Cx. quinquefasciatus*, and *Cx. restuans.* Negative correlations between adult (**black**) and immature (**red**) survival and temperatures could offset increases vectorial capacity associated with increased rates of mosquito and virus proliferation, particularly for *Cx. restuans*. Adult longevity is significantly lower for *Cx. restuans* relative to both *Cx. pipiens and Cx.* *quinquefasciatus*, which are statistically equivalent; and the effect of temperature on the percentage of larvae reaching the adult stage (emergence) is significantly greater for *Cx. restuans* relative to both other species. Adapted from [66].

Extrinsic factors are known to have a direct influence on mosquito survivorship, developmental rates of immature stages, and, subsequently, population density [61,62]. Temperature is a particularly important factor for mosquitoes as it can directly affect life history traits important in vectorial capacity [61,63,64]. For this reason, current and predicted temperature rises resulting from global climate change are likely to significantly alter patterns of WNV and other vector-borne diseases [65]. Studies with field populations of Culex mosquitoes in general indicate that increases in temperature are likely to accelerate mosquito development [62], demonstrating that increasing temperatures are associated with accelerated proliferation. A recent study confirmed these findings, yet also demonstrated that this effect is less pronounced at temperatures over 24 °C, suggesting that rising temperatures in milder regions may have lesser effects on the rate of Culex development than would increases in regions where mean summer temperatures range from 16–24 °C [66]. In addition, it was shown that a negative correlation between temperature and longevity could at times result in an overall decline in vectorial capacity despite accelerated development. This same study demonstrated that among field populations of *Cx. pipiens*, *Cx. quinquefasciatus*, and *Cx. restuans*, *Cx. restuans* are most likely to be affected by temperature increases, with increased sensitivity to temperature rises over 16 °C as evidenced by adult and immature mortality (Figure 2). Mild winter and early spring conditions may also contribute to larger springtime Culex populations and earlier commencement of WNV activity, yet abnormally high winter temperatures may result in premature exodus from hibernacula and winter mortality in diapausing populations [67,68].

The impact of larval nutrition on development and vector competence is virus- and vector-specific. Studies conducted with *Cx. tarsalis* and WNV demonstrated that nutritional deprivation of larvae increased development time, decreased pupation and emergence rates, and resulted in decreased adult female body size, yet no consistent differences in WNV infection, dissemination, and transmission rates were measured [69]. These results are similar to those with *Aedes vigilax* infected with *Ross River virus* [70], but differ from those with *La Crosse virus* (LACV) and *Ae. triseriatus* [71]. The effect of sucrose concentration on adult survivorship and vector competence was explored with *Cx. pipens* and WNV, demonstrating that survivorship was directly proportional to sucrose intake, but also that mosquitoes with lower nutrient reserves as a result of less concentrated sucrose meals were more likely to orally transmit virus [72].

The interaction between rainfall and mosquito populations is a dynamic one, and also species-specific. Decreased precipitation has been associated with increased WNV infection prevalence in horses, birds and humans [73,74,75]. Although this may be partially due to increased population density of the vector due to both increased breeding/development success and decreased predation, it appeared to be more likely due to increased activity in the mosquito population [76]. 

#### 2.2.3. Blood Feeding Behavior (a)

Mosquitoes may be generalists or opportunists in feeding, or they may be specialists with a strong preference for a single host species. Primary WNV vectors may feed on a range of avian and mammalian hosts with variable levels of WNV competence [24], therefore determining temporal and spatial variability in feeding preference and intensity is critical to predicting patterns of WNV transmission. Many geographic and species-specific differences in blood feeding behavior among Culex mosquitoes have been noted [15,77,78,79]. Effective bridge vectors of WNV feed on avian hosts during their first blood meal and humans during the second, to become infected with and transmit virus, respectively. In general, *Cx. tarsalis* tend to be more generalist feeders than the more ornithophilic *Cx. pipens* complex mosquitoes [77,79,80,81], yet temporal alterations in feeding behavior likely drive the spillover of WNV from the enzootic cycle of *Cx. pipiens*, *Cx. quinquefasciatus*, and *Cx. tarsalis* from birds early in the transmission season to humans later [82,83]. Genetic signature of *Cx. pipiens* mosquitoes may also be an important contributor to host feeding preference. Northern European populations of *Cx.* *pipiens* are pure, whereas USA populations generally contain varying levels of *Cx.* *molestus* signature, a characteristic which may correlate to geographical differences in the propensity to feed on mammals and the likelihood of spillover to humans [21,84]. Genetically determined preferences may be supplanted by availability of the preferred hosts, e.g., vectors with avian preferences will be affected by where the bird nests, what time of day the bird is active, and where it forages. Avian population distribution is a critical factor, with droughts often resulting in the aggregation of Culex species mosquitoes and wild birds, leading to high levels of WNV transmission [73]. 

### 2.3. WNV Infection and Mosquito Life History Traits

**Figure 3:**
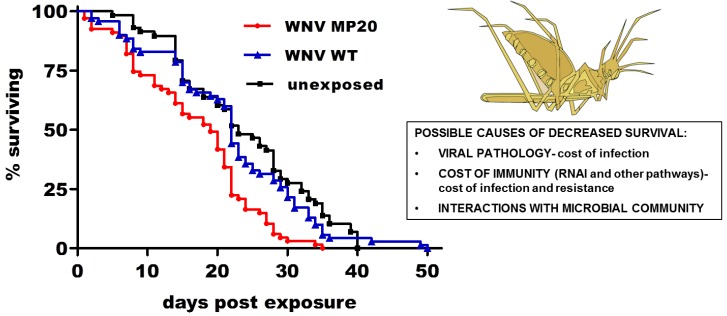
Costs of West Nile virus (WNV) infection and resistance and strain-specific effects on survival in *Culex pipiens.* Exposure to mosquito-adapted WNV (MP20) decreases survival with or without establishment of infection (costs of infection and resistance) while exposure to wildtype (WT) WNV does not alter survival. Decreased survival in infected mosquitoes decreases vectorial capacity despite increased vector competence of WNV MP20. Adapted from [89].

Although interactions between mosquito vectors and the pathogens they carry are generally characterized as avirulent, arboviral infections can at times significantly alter mosquito life-history traits and, subsequently, vectorial capacity. Recent studies with WNV have begun to demonstrate that such interactions are highly specific and, similar to vertebrate infections; the outcomes of mosquito infections are likely to be dependent on both host species and viral strain. Infection of *Cx. tarsalis* with WNV demonstrated decreased fecundity and increased blood feeding rates in infected mosquitoes which, as has been shown with Plasmodium-mosquito interactions [85] and LACV [86], could enhance vectorial capacity [87]. Subsequent studies with *Cx. pipiens* demonstrated that WNV infection is not associated with alterations to life-history traits, showing species specificity, yet that resistance to infection may be associated with decreased longevity [88]. This survival cost of resistance, which has also been demonstrated in a more recent study with WNV [89], as well as with *Dengue virus* (DENV; [90]), suggests the maintenance of a potentially costly immune response to invading arboviruses and a potential cost in vector populations resulting from arbovirus exposure alone. A follow-up study demonstrated that a WNV infection can indeed be virulent in *Cx. pipiens*, with decreased longevity as well as altered fecundity and blood feeding patterns associated with exposure to a mosquito-adapted strain but not wildtype WNV [89]. These data establish that a highly-fit WNV strain with superior intrinsic fitness may ultimately have decreased vectorial capacity due to strain-specific effects on life history traits (Figure 3). Previous studies with alphaviruses demonstrate a similar fitness cost from infection [91,92,93], as does recent work with DENV [90], yet the variability and specificity of these outcomes demonstrated with WNV adds complexity to our understanding of the role of vector-virus interactions in governing transmission intensity. The specific mechanisms of WNV virulence in mosquitoes remain uncharacterized. Studies in *Cx. quinquefasciatus* demonstrated that infection can be associated with tissue damage in salivary glands [94] and apoptosis of midgut cells [95], suggesting that virulence could be a direct result of viral pathology, yet it is also possible that there is an indirect metabolic cost resulting from activation of the immune response, as suggested by the documented costs of resistance. Regardless of the mechanisms, these studies indicate that viral strain and mosquito species-specific effects on life-history traits are important factors contributing to WNV vectorial capacity. 

### 2.4. Mosquito Immunity and Microbial Interactions

Recent studies have also advanced our understanding of the complexity of interactions between invading arboviruses and mosquito immune defenses [96]. It is now well established that the primary immune response to an arboviral infection of a mosquito is the RNA interference pathway (RNAi; [97]. RNAi produces specific, small interfering RNA (siRNA) signals that target double-stranded viral RNA for degradation and in turn modulate viral replication. This siRNA-mediated silencing has been shown to provide functional innate immunity in invertebrates against a range of RNA viruses [98], including WNV [99]. In addition, classical innate immune pathways, including IMD, Jak-STAT, and Toll have been implicated as secondary pathways in modulating WNV infection in mosquitoes [100] and activation of these pathways may be initiated by crosstalk with the RNAi response [101]. An extensive review of the interactions between flaviviruses and the mosquito innate immune responses can be found in this issue [102].

Although co-infections among WNV and other pathogenic arboviruses are rare in mosquitoes, increasing numbers of insect-specific flaviviruses (ISFs) have been identified in recent years, many in primary vectors of WNV. These include *Cell fusing agent virus* [103], *Kamiti River virus* [104], *Culex flavivirus* (CxFV; [105]), *Lammi virus* [106], *Calbertado virus* [107], and *Palm Creek virus* (PCV; [108]). The presence of co-infection with ISFs and WNV has been noted [109,110,111], yet the extent of interaction and/or effect on WNV transmissibility is not fully defined. Although epidemiological studies have identified an association between CxFV infection and WNV infection [109,110] and experimental evidence suggests co-infection could enhance WNV transmission via complementation in *Cx. quinquefasciatus* [112], recent studies link CxFV infection with delayed progression of WNV infection in *Cx. pipiens* [111], and PCV infection with suppression of WNV replication in mosquito cell culture [108], as would be predicted by the concept of super-infection exclusion [113]. Together, these data suggest that the relationship between WNV transmission and ISF infections are also likely to be variable and species-specific, perhaps as a result of the extent of evolutionary history between these viruses and individual mosquito populations.

Although it is still unclear if the extent or effect of co-infection with ISFs is extensive enough to significantly alter vector-WNV interactions in nature, microbial infections of mosquitoes are ubiquitous, and interactions with bacterial flora are known to directly impact arbovirus infection and replication [114]. A recent trial to exploit this symbiont-mediated protection utilizing introduction of maternally inherited bacteria of the genus *Wolbachia* for the control of DENV in *Ae. aegypti* in northern Australia was successfully carried out [115]. The primary mechanism of virus inhibition of these bacteria is likely activation of the Toll pathway via induction of reactive oxygen species [116]. *Wolbachia* spp. are native to the WNV vectors *Cx. pipiens* and *Cx. quinquefasciatus*, but not *Cx. tarsalis*, and have been found to increase resistance to WNV infection in the laboratory [117]. Not surprisingly, additional studies have also found the capacity of *Wolbachia* to modulate WNV infection is both strain and species-specific *in vitro* [118], as has been shown with DENV [119]. It is unlikely from an evolutionary perspective that the capacity to modulate viral infection would be limited to a single bacterial species, particularly given recent advances in understanding the redundancy of mosquito immune pathways [96]. Indeed, many more bacterial genera can likely be found in primary vectors of WNV [120], and studies with DENV suggest complex interactions between the gut microbiome, innate immunity, and vector competence [121]. In addition to direct interactions with viral infection and replication, microbial populations may also alter life-history traits, including feeding behavior, which itself could lead to significant variation in vectorial capacity independent of direct effects on vector competence [122]. Furthermore, many fungal species inhabit mosquitoes [123,124] and a recent study with *Ae. aegypti* demonstrates significantly reduced vectorial capacity for DENV-2 resulting from co-infection with the fungus *M. anisopliae* [125].

## 3. Vertebrate

WNV is zoonotic and may be considered an ecological generalist, as not only can the virus be transmitted by numerous species of mosquitoes, it also is capable of infecting numerous and diverse vertebrate hosts. Reservoir competence, *i.e.*, an index that reflects the relative number of infectious mosquitoes that would be derived from feeding on each host species, is a useful measure of the relative importance of each vertebrate species. This value depends on the susceptibility of the vertebrate host, the concentration of infectious virus particles in the blood, and the duration of an infectious level viremia in the host [126]. 

### 3.1. Avian Host Competence

The enzootic transmission cycle is comprised predominantly by ornithophilic Culex species and passeriform birds. West Nile virus causes neurologic disease in some avian species, particularly passeriforms, while other species demonstrate variable levels of morbidity and mortality as demonstrated in laboratory studies [127] and in serosurveys [128]. The high mortality observed in wild birds in the USA [129] is unique to North America, but mortality was noted in birds in Israel in 1998, and hooded crows experimentally infected in Egypt [130]. The *Corvidae* (Order *Passeriformes*) are among the most susceptible avian families to WN disease [127,131], but the virus has caused death and morbidity in at least 326 species [132]. A serosurvey of American crows in 1999 in New Jersey found 5.1% seropositive for WNV [133], and 50% of wild birds tested in the epicenter (Queens, NY, USA) were seropositive [134]. Nonetheless, severe regional declines have been noted in some avian species [131] following introduction of WNV. The increased avian mortality from WNV in the U.S. likely increases the intensity of transmission (*i.e.*, R_0_) in the enzootic cycle partly by removing individuals who would have become immune. This increase in enzootic R_0_ might be expected to result in more human infections [24].

Migratory birds are thought to have been the principal agent for the spread of WNV in North America and globally. Infectious viremias were detected in birds during fall migration in 2002 and 2003 [128]. In addition, viremic white storks (*Ciconia ciconia*) in Israel were identified during migration within two days of arrival at a stopover site [135]. 

### 3.2. Other Vertebrates

Although the main enzootic vectors, Culex species mosquitoes, are ornithophilic, it has been noted that late in the transmission season they become more opportunistic and largely switch to feeding on mammals [82,136,137]. Experimental and field studies have demonstrated that like birds [127], non-avian vertebrates vary in their morbidity, mortality, host competence [138,139], as well as seroprevalence in nature [140]. Mammals, including horses and humans, are generally considered dead-end hosts, as the level of viremia they mount is too low and the length too brief to infect mosquitoes. Exceptions have been noted following laboratory studies, including tree squirrels (Sciurus spp.), eastern gray squirrels (*Sciurus carolinensis*) [139] eastern chipmunks (*Tamias striatus*), and eastern cottontail rabbits (*Sylvilagus floridanus*), which develop viremia sufficient for infecting some mosquito species, but it is not clear whether the infected mosquitoes are competent, *i.e.*, will transmit virus. These studies are well summarized elsewhere and therefore will not be discussed further [141].

## 4. Virus

### 4.1. Population Genetics and Molecular Epidemiology

**Figure 4:**
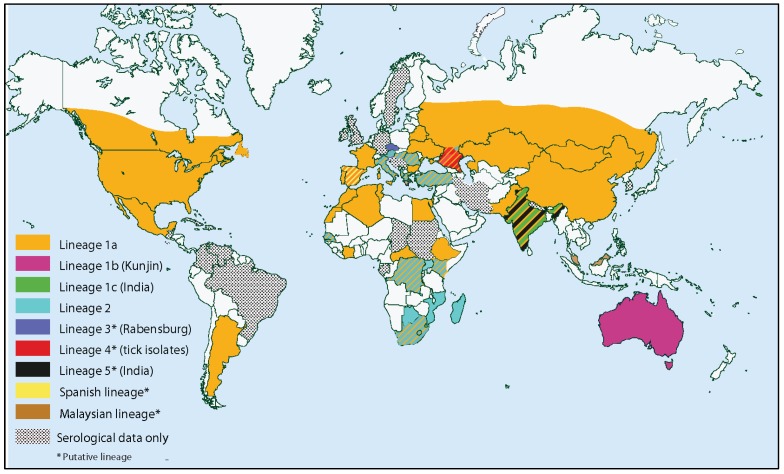
Worldwide distribution of West Nile virus.

WNV, first isolated in 1937 from a febrile patient in Uganda [142], is a member of the Japanese encephalitis serogroup and, as such, is a close relative of *Japanese encephalitis virus*, *Murray Valley encephalitis virus*, *Usutu virus*, and *St. Louis encephalitis virus* (SLEV), among others. WNV has historically been classified into at least two phylogenetically distinct lineages. Lineage 1 WNV, which is associated with West Nile fever in humans and CNS infection in approximately 1% of cases [24], is the most widely distributed, occurring in Africa, Asia, Europe, Australia and North and Central America [2]. Lineage 1 WNV can be further divided into three sublineages: Lineage 1a is the most widely distributed, occurring in Africa, Europe and the Americas; lineage 1b, also known as Kunjin virus, occurs in Australia; lineage 1c occurs in India. Lineage 2 is ~20% genetically divergent from lineage I, occurs in sub-Saharan Africa, where it is the cause of WNF [143]. Although once thought to be incapable of invading the CNS, recent evidence of neuoroinvasive disease resulting from infection with Lineage 2 WNV has emerged [144,145] and some Lineage 2 strains are virulent in mice [146]. Rabensburg virus (RabV), which has 75%–77% nucleotide identity and 89%–90% amino acid identity with representative members of Lineage 1 and 2 WNV, was isolated from *Cx. pipiens* in the Czech Republic in 1997 [147] and subsequently from *Aedes rossicus* Dolbescin, Gorickaja and Mitrofanova [148]. Initially considered a Lineage 3 WNV, RabV is limited in its capacity to infect vertebrate hosts and may therefore represent an intermediate between a mosquito-specific and horizontally-transmitted flavivirus [149]. Further antigenic and genetic analyses of isolates from Russia, India and Malaysia suggest that WNV could in fact be classified into seven distinct lineages [150,151,152,153]. Charrel *et al*. [154] initially defined membership in the species WNV to be <21% genetic distance, yet inclusion of Lineages 3–7 would result in sequence divergences of over 25% for some isolates (Figure 4; [152]).

Sequence analyses following the WNV U.S. invasion are consistent with a single point introduction with a strain most closely related to a 1998 Israeli strain [2] which had likely been introduced to Israel by migrating storks from Africa [135,155]. Despite subsequent range expansion throughout the Americas, WNV has remained relatively genetically static and unstructured, although some recent evidence for geographic structure has emerged [156,157]. The most phylogenetically distinct event occurring in the U.S. was the complete displacement of the introduced strain by WN02, a genotype distinguished by just a single consistent amino acid change in the envelope protein, V159A, which likely drove displacement by decreasing EIP in Culex mosquitoes [50,52], facilitated by warmer than average temperatures [51]. There is also evidence for spread of another distinct genotype, SW/WN03, characterized my amino acid changes in the NS4B and NS5 coding regions [156]. Although rare, other evidence of WNV adaptive change in the U.S. exists, including NS3 and NS4a mutations associated with increased virulence in birds [157,158], as well as positive selection acting on other genes including E, NS2A and NS5 [143,156,159].

### 4.2. Bottlenecks and Intrahost Diversity

The commencement of infection in the mosquito mesenteron (midgut) is likely constrained to a limited number of cells in the posterior midgut epethelia [7,160] and therefore often imposes a significant genetic bottleneck on host-derived viral populations [161,162]. Although this bottleneck is documented in both *Cx. pipiens* [161] and *Cx. quinquefasciatus* [162], additional genetic bottlenecks have been identified with salivary gland infection and transmission in *Cx. pipiens* but not *Cx. quinquefasciatus*, consistent with species-specific differences in competence [45]*.* In addition, significant temporal decreases in intrahost diversity have been identified [161,162], particularly in tissues responsible for secondary amplification, suggesting a stochastic narrowing of the swarm possibly driven by a decrease in the extent of infected cells over time as mosquito innate immune pathways are triggered. In addition to these stochastic events, positive selection is also likely to occur at times, both across barriers and through time. WNV intrahost populations may overcome the negative effects of genetic narrowing through high mutation rates shared among RNA viruses [163] in concert with relaxed purifying selection [164]. Consistent with this, intrahost diversity of mosquito-derived WNV has generally been shown to be more extensive than avian-derived WNV [165,166,167], similar to what has been shown for SLEV; [168]. This genetic diversity likely corresponds to phenotypic diversity [169] and therefore host breadth [170], and may also be advantageous to replicative fitness in mosquitoes due to an increased capacity to evade the mosquito RNAi response [171,172]. Despite this, experimental evolution studies in *Cx. pipiens*, which utilized passage of virus-positive salivary secretions, demonstrate that a combination of selection and intrahost bottlenecks may purge diversity, yet this does not necessarily preclude WNV from adapting further to mosquito hosts [173]. 

Although more extensive purifying selection within avian hosts serves to decrease WNV intrahost diversity [164,167], genetic bottlenecks within birds have not been well defined. It is likely that significant narrowing of the viral swarm could occur with neouroinvasive infections, yet the production of infectious viremia levels alone does not itself require crossing extensive barriers; therefore stochastic events in the avian host may not contribute as significantly to shaping the WNV swarm as those encountered during mosquito infections. 

As the intensity of WNV transmission cycles in concert with *Culex* populations, seasonal genetic bottlenecks are also likely, particularly in temperate areas in which mosquitoes must overwinter. Diapausing *Cx. pipiens* and *Cx. tarsalis*, likely infected via vertical transmission [174,175,176], are the most probable candidates to serve as winter reservoirs for WNV, a maintenance process which may impose substantial declines in WNV inter- and intrahost genetic variability, particularly because rates of vertical transmission, as well as successful transtadial transmission, are low [177,178]. The extent of this bottleneck may depend on the success of overwintering populations, which is governed largely by variation in winter temperatures [68]. Although the genetic and phenotypic consequences of vertical transmission or temporal variation in WNV swarm breadth have not been evaluated, studies with SLEV demonstrate swarm breadth may decrease over time, which could increase vulnerability to the negative consequences of genetic isolation [179]. Given differences in transmission intensity and levels of activity between WNV and SLEV, seasonal bottlenecks may prove less of a threat to WNV, as the rapid and widespread local diversification of WNV swarms may generate sufficiently high levels of diversity prior to subsequent breaks in transmission. 

### 4.3. Adaptive Constraint

Although recent phylogenetic analyses have revealed some instances of positive selection [159], and adaptive change has been noted with the displacement of the NY99 strain with WNV02 [50], WNV, like many arboviruses, remains relatively genetically and phenotypically conserved despite the enormous adaptive potential that accompanies rapid replication of RNA genomes [1,180,181]. Such constraint is historically attributed to the cost of cycling between disparate hosts [182,183]. Specifically, a cost for host-specific adaptation in the alternate host may slow or prevent fixing of beneficial mutations, an idea evolutionarily synonymous to antagonistic pleiotropy [184]. Evidence for such adaptive tradeoffs for WNV is mixed, with no fitness cost in vertebrate cells resulting from adaptation to mosquito cell culture [185] or in terms of viremia kinetics in chicks resulting from mosquito adaptation achieved through sequential passage of *Cx. pipiens* salivary secretions [173]. In contrast, sequential passage of WNV derived from whole *Cx. pipiens* bodies may result in a loss of fitness in chicks, yet, interestingly, passage and adaptation to chicks may simultaneously increase WNV fitness in mosquitoes [186]. These data demonstrate, not surprisingly, that adaptive trade-offs may occur, but are not the rule for WNV, which is clearly a generalist capable of high levels of co-adaptation. 

Theory predicts that under high mutation rates in which selection cannot outpace mutation, RNA virus evolution should favor mutational robustness to avoid accumulation of deleterious mutations [187]. Such robustness may effectively trap arboviruses in sub-optimal fitness landscapes, such that mutational neighbors have a tendency to be phenotypically equivalent. This scenario implies that these viruses may often require multiple, epistatic mutations to attain fitness gains, an idea that is supported by evolutionary and adaptive change noted in alphaviruses including *Chikungunya virus* [188] and *Venezuelan equine encephalitis virus* [189]. An evolutionary strategy which could overcome the need for mutational robustness and promote phenotypic diversity is widespread complementation and cooperative interactions among co-infecting strains. WNV can evolve to overcome super-infection exclusion [113], and may readily share gene products in co-infected cells. A recent study demonstrates that evolution of a WNV swarm in the presence of co-infection promotes complementation which acts to maintain genetic and phenotypic diversity. Furthermore, although such interactions may slow selection for high fitness variants, cooperation may result in swarm fitness levels which exceed that of any individual [169]. Although the extent of co-infection and/or cooperative or inhibitory interactions *in vivo* remain(s) uncharacterized, studies with WNV-infected *Cx. quinquefasciatus* suggest that complementation could act to maintain deletion mutants [162], as has been shown with DENV in *Ae. aegypti* [190], demonstrating that the capacity for frequent interactions among viable particles *in vivo* exists and could contribute significantly to WNV swarm dynamics, adaptation, and evolvability. 

## 5. Concluding Remarks

Patterns and intensity of WNV transmission are governed by complex and dynamic interactions between vector, vertebrate, virus and environment. The capacity to characterize the role of extrinsic and intrinsic factors that determine the outcomes of these interactions is complicated by the fact that effects may rarely act independently. Instead, as has been shown with DENV [191,192], it is likely that vector-virus genotype by genotype interactions, as well as genotype by environment interactions are the rule. This confounds our ability to provide static, generic characterization of the transmission potential of individual WNV genotypes or mosquito populations, yet this variability also provides an opportunity to more completely uncover the mechanisms that govern WNV transmission. While the historic focus with regards to vector-WNV interactions has been on assessing variation in vector competence, since both species and strain-specific effects on life-history traits are now evident [88,89], a shift towards considering the whole entity of WNV vectorial capacity is required if we are to accurately bridge virus and vector genotypes to transmission potential. On the virus side, a more in-depth understanding of the phenotypic importance of the WNV swarm, as well as the selective pressures that shape these swarms, is required. Characterizing interactions within viral swarms as well as between WNV and both microbial populations and host immune genes should be a focus of future studies. In particular, further characterization of important genes and immune pathways associated with WNV infection of mosquitoes, as well as defining the physiological costs of infection and resistance are important to better define how host variation and evolution guide patterns of transmission. In regard to extrinsic factors, continued efforts to define how environmental fluctuations work in concert with host and viral variation to alter transmission intensity is critical, particularly at a time when worldwide climatic shifts are likely to dramatically alter environmental and ecological landscapes, and anthropogenic change is rampant. Although many predicted that WNV activity in the U.S. was likely to continue to equilibrate or decline following a peak in 2003 [165,193], unprecedented activity occurred in 2012 in terms of both confirmed neuroinvasive cases and deaths [194]. Preliminary evidence suggests it is likely environmental change, rather than genetic change, in the virus triggered this spike in WNV activity [195], yet the specific factors driving this resurgence remain undefined. This is a reminder that, despite over a decade of high quality WNV research, further studies are required to fully understand the specific factors that dictate patterns of WNV transmission. 

## References

[1] Ebel G.D., Dupuis A.P., Ngo K.A., Nicholas D.C., Kauffman E.B., Jones S.A., Young D.M., Maffei J.G., Shi P.-Y., Bernard K.A. (2001). Partial genetic characterization of West Nile virus strains, New York State. Emerg. Infect. Dis..

[2] Lanciotti R.S., Roehrig J.T., Deubel V., Smith J., Parker M., Steele K., Crise B., Volpe K.E., Crabtree M.B., Scherret J.H. (1999). Origin of the West Nile virus responsible for an outbreak of encephalitis in the northeastern United States. Science.

[3] Bosch I., Herrera F., Navarro J.C., Lentino M., Dupuis A.P., Maffei J., Jones M.J., Fernandez E., Perez N., Perez-Eman J. (2007). West Nile viurs, Venezuela. Emerg. Infect. Dis..

[4] Hayes E.B., Sejvar J.J., Zaki S.R., Lanciotti R.S., Bode A.V., Campbell G.L. (2005). Virology, pathology, and clinical manifestations of West Nile virus disease. Emerg. Infect. Dis..

[5] Komar N., Clark G.G. (2006). West Nile virus activity in Latin America and the Caribbean. Rev. Panam. Salud Publica.

[6] Morales M.A., Barrandeguy M., Fabbri C., Garcia J.B., Vissani A., Trono K., Gutierrez G., Pigretti S., Menchaca H., Garrido N. (2006). West Nile virus isolation from equines in Argentina, 2006. Emerg. Infect. Dis..

[7] Chamberlain R.W., Sudia W.D. (1961). Mechanism of transmission of viruses by mosquitoes. Annu. Rev. Entomol..

[8] Hardy J.L., Houk E.J., Kramer L.D., Reeves W.C. (1983). Intrinsic factors affecting vector competence of mosquitoes for arboviruses. Annu. Rev. Entomol..

[9] Macdonald G. (1961). Epidemiologic models in studies of vector-borne diseases. Public Health Rep..

[10] Kramer L.D., Ebel G.D. (2003). Dynamics of flavivirus infection in mosquitoes. Adv. Virus Res..

[11] Reiner R.C., Perkins T.A., Barker C.M., Niu T., Chaves L.F., Ellis A.M., George D.B., Le M.A., Pulliam J.R., Bisanzio D. (2013). A systematic review of mathematical models of mosquito-borne pathogen transmission: 1970–2010. J. R. Soc. Interface.

[12] Kilpatrick A.M., Kramer L.D., Campbell S.R., Alleyne E.O., Dobson A.P., Daszak P. (2005). West Nile virus risk assessment and the bridge vector paradigm. Emerg. Infect. Dis..

[13] Ebel G.D., Rochlin I., Longacker J., Kramer L.D. (2005). *Culex restuans* (Diptera: Culicidae) relative abundance and vector competence for West Nile virus. J. Med. Entomol..

[14] Geery P.R., Holub R.E. (1989). Seasonal abundance and control of Culex spp. in catch basins in Illinois. J. Am. Mosq. Control Assoc..

[15] Savage H.M., Aggarwal D., Apperson C.S., Katholi C.R., Gordon E., Hassan H.K., Anderson M., Charnetzky D., McMillen L., Unnasch E.A. (2007). Host choice and West Nile virus infection rates in blood-fed mosquitoes, including members of the *Culex pipiens* complex, from Memphis and Shelby County, Tennessee, 2002–2003. Vector Borne Zoonotic Dis..

[16] Andreadis T.G., Anderson J.F., Vossbrinck C.R. (2001). Mosquito surveillance for West Nile virus in Connecticut, 2000: Isolation from *Culex pipiens*, *Cx. restuans*, *Cx. salinarius*, and *Culiseta melanura*. Emerg. Infect. Dis..

[17] Fonseca D.M., Okada K., Kramer L.D. (2009). Microsatellite loci for the white-dotted mosquito (Culex restuans), a principal vector of West Nile virus in North America. Mol. Ecol. Resour..

[18] Collins F.H., Paskewitz S.M. (1996). A review of the use of ribosomal DNA (rDNA) to differentiate among cryptic Anopheles species. Insect Mol. Biol..

[19] Harbach R.E., Dahl C., White G.B. (1985). *Culex* (Culex) *pipiens* Linnaeus (Diptera: Culicidae): Concepts, type designations, and description. Proc. Entomol. Soc. Wash..

[20] Fonseca D.M., Smith J.L., Kim H.C., Mogi M. (2009). Population genetics of the mosquito *Culex pipiens* pallens reveals sex-linked asymmetric introgression by Culex quinquefasciatus. Infect. Genet. Evol..

[21] Fonseca D.M., Keyghobadi N., Malcolm C.A., Mehmet C., Schaffner F., Mogi M., Fleischer R.C., Wilkerson R.C. (2004). Emerging vectors in the Culex pipiens complex. Science.

[22] Farajollahi A., Fonseca D.M., Kramer L.D., Marm K.A. (2011). “Bird biting” mosquitoes and human disease: A review of the role of *Culex pipiens* complex mosquitoes in epidemiology. Infect. Genet. Evol..

[23] Andreadis T.G. (2012). The contribution of Culex pipiens complex mosquitoes to transmission and persistence of West Nile virus in North America. J. Am. Mosq. Control Assoc..

[24] Kramer L.D., Styer L.M., Ebel G.D. (2007). A global perspective on the epidemiology of West Nile virus. Annu. Rev. Entomol..

[25] Huang S., Molaei G., Andreadis T.G. (2011). Reexamination of *Culex pipiens* hybridization zone in the Eastern United States by ribosomal DNA-based single nucleotide polymorphism markers. Am. J. Trop. Med. Hyg..

[26] Kothera L., Zimmerman E.M., Richards C.M., Savage H.M. (2009). Microsatellite characterization of subspecies and their hybrids in *Culex pipiens* complex (Diptera: Culicidae) mosquitoes along a north-south transect in the central United States. J. Med. Entomol..

[27] Sanogo Y.O., Kim C.H., Lampman R., Halvorsen J.G., Gad A.M., Novak R.J. (2008). Identification of male specimens of the *Culex pipiens* complex (Diptera: Culicidae) in the hybrid zone using morphology and molecular techniques. J. Med. Entomol..

[28] Ciota A.T., Chin P.A., Kramer L.D. (2013). The effect of hybridization of *Culex pipiens* complex mosquitoes on transmission of *West Nile virus*. Parasites Vectors.

[29] Brown H.E., Childs J.E., Diuk-Wasser M.A., Fish D. (2008). Ecological factors associated with West Nile virus transmission, northeastern United States. Emerg. Infect. Dis..

[30] Kilpatrick A.M. (2011). Globalization, land use, and the invasion of West Nile virus. Science.

[31] Kent R., Harrington L., Norris D. (2007). Genetic differences between *Culex pipiens* f. molestus and *Culex pipiens* pipiens (Diptera: Culicidea) in New York. J. Med. Entomol..

[32] Harbach R.E. (2012). Culex pipiens: Species versus species complex taxonomic history and perspective. J. Am. Mosq. Control Assoc..

[33] Bolling B.G., Moore C.G., Anderson S.L., Blair C.D., Beaty B.J. (2007). Entomological studies along the Colorado Front Range during a period of intense West Nile virus activity. J. Am. Mosq. Control Assoc..

[34] Reisen W.K., Reeves W.C., Reeves W.C. (1990). Bionomics and Ecology of *Culex tarsalis* and Other Potential Mosquito Vector Species. Epidemiology and Control of Mosquito-borne Arboviruses in California, 1943–1987.

[35] Goldberg T.L., Anderson T.K., Hamer G.L. (2010). West Nile virus may have hitched a ride across the Western United States on Culex tarsalis mosquitoes. Mol. Ecol..

[36] Bolling B.G., Barker C.M., Moore C.G., Pape W.J., Eisen L. (2009). Seasonal patterns for entomological measures of risk for exposure to Culex vectors and West Nile virus in relation to human disease cases in northeastern Colorado. J. Med. Entomol..

[37] Bowden S.E., Magori K., Drake J.M. (2011). Regional differences in the association between land cover and West Nile virus disease incidence in humans in the United States. Am. J. Trop. Med. Hyg..

[38] Venkatesan M., Rasgon J.L. (2010). Population genetic data suggest a role for mosquito-mediated dispersal of West Nile virus across the western United States. Mol. Ecol..

[39] Venkatesan M., Westbrook C.J., Hauer M.C., Rasgon J.L. (2007). Evidence for a population expansion in the West Nile virus vector Culex tarsalis. Mol. Biol. Evol..

[40] Kramer L.D., Hardy J.L., Presser S.B., Houk E.J. (1981). Dissemination barriers for western equine encephalomyelitis virus in *Culex tarsalis* infected after ingestion of low viral doses. Am. J. Trop. Med. Hyg..

[41] Grimstad P.R., Paulson S.L., Craig G.B. (1985). Vector competence of *Aedes hendersoni* (Diptera: Culicidae) for La Crosse virus and evidence of a salivary-gland escape barrier. J. Med. Entomol..

[42] Miller B.R., Monath T.P., Tabachnick W.J., Ezike V.I. (1989). Epidemic yellow fever caused by an incompetent mosquito vector. Trop. Med. Parasitol..

[43] Garrett-Jones C. (1964). Prognosis for interruption of malaria transmission through assessment of the mosquito’s vectorial capacity. Nature.

[44] Macdonald G. (1957). The Epidemiology and Control of Malaria.

[45] Turell M.J., Dohm D.J., Sardelis M.R., Oguinn M.L., Andreadis T.G., Blow J.A. (2005). An update on the potential of north American mosquitoes (Diptera: Culicidae) to transmit West Nile Virus. J. Med. Entomol..

[46] Goddard L.B., Roth A.E., Reisen W.K., Scott T.W. (2002). Vector competence of California mosquitoes for West Nile virus. Emerg. Infect. Dis..

[47] Turell M.J., O’Guinn M.L., Dohm D.J., Jones J.W. (2001). Vector competence of North American mosquitoes (Diptera: Culicidae) for West Nile virus. J. Med. Entomol..

[48] Turell M.J., O’Guinn M., Oliver J. (2000). Potential for New York mosquitoes to transmit West Nile virus. Am. J. Trop. Med. Hyg..

[49] Sardelis M.R., Turell M.J., Dohm D.J., O’Guinn M.L. (2001). Vector competence of selected North American Culex and *Coquillettidia mosquitoes* for West Nile virus. Emerg. Infect. Dis..

[50] Moudy R.M., Meola M.A., Morin L.L., Ebel G.D., Kramer L.D. (2007). A newly emergent genotype of west nile virus is transmitted earlier and more efficiently by culex mosquitoes. Am. J. Trop. Med. Hyg..

[51] Kilpatrick A.M., Meola M.A., Moudy R.M., Kramer L.D. (2008). Temperature, viral genetics, and the transmission of West Nile virus by Culex pipiens mosquitoes. PLoS Pathog..

[52] Ebel G.D., Carricaburu J., Young D., Bernard K.A., Kramer L.D. (2004). Genetic and phenotypic variation of West Nile virus in New York, 2000–2003. Am. J. Trop. Med. Hyg..

[53] Dohm D.J., O’Guinn M.L., Turell M.J. (2002). Effect of environmental temperature on the ability of Culex pipiens (Diptera: Culicidae) to transmit West Nile virus. J. Med. Entomol..

[54] Reisen W.K., Fang Y., Martinez V.M. (2006). Effects of temperature on the transmission of west nile virus by *Culex tarsalis* (Diptera: Culicidae). J. Med. Entomol..

[55] Reisen W.K., Barker C.M., Fang Y., Martinez V.M. (2008). Does variation in Culex (Diptera: Culicidae) vector competence enable outbreaks of West Nile virus in California?. J. Med. Entomol..

[56] Sardelis M.R., Turell M.J., O’Guinn M.L., Andre R.G., Roberts D.R. (2002). Vector competence of three North American strains of *Aedes albopictus* for West Nile virus. J. Am. Mosq. Control Assoc..

[57] Vaidyanathan R., Scott T.W. (2007). Geographic variation in vector competence for West Nile virus in the *Culex pipiens* (Diptera: Culicidae) complex in California. Vector Borne Zoonotic Dis..

[58] Hayes C.G., Baker R.H., Baqar S., Ahmed T. (1984). Genetic variation for West Nile virus susceptibility in *Culex tritaeniorhynchus*. Am. J. Trop. Med. Hyg..

[59] Vaidyanathan V., Scott T.W. (2006). Seasonal variation in susceptibility to West Nile virus infection in *Culex pipiens* pipiens (L.) (Diptera: Culicidea) from San Joaquin County, California. J. Vector Ecol..

[60] Kilpatrick A.M., Fonseca D.M., Ebel G.D., Reddy M.R., Kramer L.D. (2010). Spatial and temporal variation in vector competence of *Culex pipiens* and *Cx. restuans* mosquitoes for West Nile virus. Am. J. Trop. Med. Hyg..

[61] Delatte H., Gimonneau G., Triboire A., Fontenille D. (2009). Influence of temperature on immature development, survival, longevity, fecundity, and gonotrophic cycles of Aedes albopictus, vector of chikungunya and dengue in the Indian Ocean. J. Med. Entomol..

[62] Rueda L.M., Patel K.J., Axtell R.C., Stinner R.E. (1990). Temperature-dependent development and survival rates of *Culex quinquefasciatus* and *Aedes aegypti* (Diptera: Culicidae). J. Med. Entomol..

[63] Dye C. (1992). The analysis of parasite transmission by bloodsucking insects. Annu. Rev. Entomol..

[64] Su T., Mulla M.S. (2001). Effects of temperature on development, mortality, mating and blood feeding behavior of *Culiseta incidens* (Diptera: Culicidae). J. Vector Ecol..

[65] Kilpatrick A.M., Randolph S.E. (2012). Drivers, dynamics, and control of emerging vector-borne zoonotic diseases. Lancet.

[66] Ciota A.T., Matacchiero A.C., Kilpatrick A.M., Kramer L.D. The effect of temperature on life history traits of *Culex* mosquitoes. J. Med. Entomol..

[67] Reisen W.K., Thiemann T., Barker C.M., Lu H., Carroll B., Fang Y., Lothrop H.D. (2010). Effects of warm winter temperature on the abundance and gonotrophic activity of *Culex* (Diptera: Culicidae) in California. J. Med. Entomol..

[68] Ciota A.T., Drummond C.L., Drobnack J., Ruby M.A., Kramer L.D., Ebel G.D. (2011). Emergence of *Culex pipiens* from overwintering hibernacula. J. Am. Mosq. Control Assoc..

[69] Dodson B.L., Kramer L.D., Rasgon J.L. (2011). Larval nutritional stress does not affect vector competence for West Nile virus (WNV) in Culex tarsalis. Vector Borne Zoonotic Dis..

[70] Jennings C.D., Kay B.H. (1999). Dissemination barriers to Ross River virus in Aedes vigilax and the effects of larval nutrition on their expression. Med. Vet. Entomol..

[71] Grimstad P.R., Walker E.D. (1991). *Aedes triseriatus* (Diptera: Culicidae) and La Crosse virus. IV. Nutritional deprivation of larvae affects the adult barriers to infection and transmission. J. Med. Entomol..

[72] Vaidyanathan R., Fleisher A.E., Minnick S.L., Simmons K.A., Scott T.W. (2008). Nutritional stress affects mosquito survival and vector competence for West Nile virus. Vector Borne Zoonotic Dis..

[73] Shaman J., Day J.F., Stieglitz M. (2005). Drought-induced amplification and epidemic transmission of West Nile virus in southern Florida. J. Med. Entomol..

[74] Epstein P.R. (2001). West Nile virus and the climate. J. Urban Health.

[75] Johnson B.J., Sukhdeo M.V. (2013). Drought-induced amplification of local and regional West Nile virus infection rates in New Jersey. J. Med. Entomol..

[76] Crowder D.W., Dykstra E.A., Brauner J.M., Duffy A., Reed C., Martin E., Peterson W., Carriere Y., Dutilleul P., Owen J.P. (2013). West nile virus prevalence across landscapes is mediated by local effects of agriculture on vector and host communities. PLoS One.

[77] Kilpatrick A.M., Daszak P., Jones M.J., Marra P.P., Kramer L.D. (2006). Host heterogeneity dominates West Nile virus transmission. Proc. Biol. Sci..

[78] Kent R., Juliusson L., Weissmann M., Evans S., Komar N. (2009). Seasonal blood-feeding behavior of *Culex tarsalis* (Diptera: Culicidae) in Weld County, Colorado, 2007. J. Med. Entomol..

[79] Thiemann T.C., Lemenager D.A., Kluh S., Carroll B.D., Lothrop H.D., Reisen W.K. (2012). Spatial variation in host feeding patterns of *Culex tarsalis* and the *Culex pipiens* complex (Diptera: Culicidae) in California. J. Med. Entomol..

[80] Reisen W.K., Hardy J.L., Reeves W.C., Presser S.B., Milby M.M., Meyer R.P. (1990). Persistence of mosquito-borne viruses in Kern County, California, 1983–1988. Am. J. Trop. Med. Hyg..

[81] Hamer G.L., Kitron U.D., Goldberg T.L., Brawn J.D., Loss S.R., Ruiz M.O., Hayes D.B., Walker E.D. (2009). Host selection by Culex pipiens mosquitoes and West Nile virus amplification. Am. J. Trop. Med. Hyg..

[82] Kilpatrick A.M., Kramer L.D., Jones M.J., Marra P.P., Daszak P. (2006). West Nile virus epidemics in North America are driven by shifts in mosquito feeding behavior. PLoS Biol..

[83] Thiemann T.C., Wheeler S.S., Barker C.M., Reisen W.K. (2011). Mosquito host selection varies seasonally with host availability and mosquito density. PLoS Negl. Trop. Dis..

[84] Huang S., Hamer G.L., Molaei G., Walker E.D., Goldberg T.L., Kitron U.D., Andreadis T.G. (2009). Genetic variation associated with mammalian feeding in *Culex pipiens* from a West Nile virus epidemic region in Chicago, Illinois. Vector Borne Zoonotic Dis..

[85] Schwartz A., Koella J.C. (2001). Trade-offs, conflicts of interest and manipulation in *Plasmodium*-mosquito interactions. Trends Parasitol..

[86] Jackson B.T., Brewster C.C., Paulson S.L. (2012). La Crosse virus infection alters blood feeding behavior in *Aedes triseriatus* and *Aedes albopictus* (Diptera: Culicidae). J. Med. Entomol..

[87] Styer L.M., Meola M.A., Kramer L.D. (2007). West Nile virus infection decreases fecundity of *Culex tarsalis* females. J. Med. Entomol..

[88] Ciota A.T., Styer L.M., Meola M.A., Kramer L.D. (2011). The costs of infection and resistance as determinants of West Nile virus susceptibility in *Culex mosquitoes*. BMC Ecol..

[89] Ciota A.T., Ehrbar D.J., Matacchiero A.C., van Slyke G.A., Kramer L.D. (2013). The evolution of virulence of West Nile virus in a mosquito vector: Implications for arbovirus adaptation and evolution. BMC Evol. Biol..

[90] Maciel-de-Freitas R., Koella J.C., Lourenco-de-Oliveira R. (2011). Lower survival rate, longevity and fecundity of *Aedes aegypti* (Diptera: Culicidae) females orally challenged with dengue virus serotype 2. Trans. R. Soc. Trop. Med. Hyg..

[91] Mahmood F., Reisen W.K., Chiles R.E., Fang Y. (2004). Western equine encephalomyelitis virus infection affects the life table characteristics of *Culex tarsalis* (Diptera: Culicidae). J. Med. Entomol..

[92] Moncayo A.C., Edman J.D., Turell M.J. (2000). Effect of eastern equine encephalomyelitis virus on the survival of *Aedes albopictus*, *Anopheles quadrimaculatus*, and *Coquillettidia perturbans* (Diptera: Culicidae). J. Med. Entomol..

[93] Scott T.W., Lorenz L.H. (1998). Reduction of Culiseta melanura fitness by eastern equine encephalomyelitis virus. Am. J. Trop. Med. Hyg..

[94] Girard Y.A., Popov V., Wen J., Han V., Higgs S. (2005). Ultrastructural study of West Nile virus pathogenesis in *Culex pipiens* quinquefasciatus (Diptera: Culicidae). J. Med. Entomol..

[95] Vaidyanathan R., Scott T.W. (2006). Apoptosis in mosquito midgut epithelia associated with West Nile virus infection. Apoptosis.

[96] Fragkoudis R., Attarzadeh-Yazdi G., Nash A.A., Fazakerley J.K., Kohl A. (2009). Advances in dissecting mosquito innate immune responses to arbovirus infection. J. Gen. Virol..

[97] Blair C.D. (2011). Mosquito RNAi is the major innate immune pathway controlling arbovirus infection and transmission. Future Microbiol..

[98] Wang X.H., Aliyari R., Li W.X., Li H.W., Kim K., Carthew R., Atkinson P., Ding S.W. (2006). RNA interference directs innate immunity against viruses in adult Drosophila. Science.

[99] Chotkowski H.L., Ciota A.T., Jia Y., Puig-Basagoiti F., Kramer L.D., Shi P.Y., Glaser R.L. (2008). West Nile virus infection of *Drosophila melanogaster* induces a protective RNAi response. Virology.

[100] Arjona A., Wang P., Montgomery R.R., Fikrig E. (2011). Innate immune control of West Nile virus infection. Cell. Microbiol..

[101] Paradkar P.N., Trinidad L., Voysey R., Duchemin J.B., Walker P.J. (2012). Secreted Vago restricts West Nile virus infection in *Culex mosquito* cells by activating the Jak-STAT pathway. Proc. Natl. Acad. Sci. USA.

[102] Prasad A.N., Brackney D.E., Ebel G.D. The role of innate immunity in conditioning mosquito susceptibility to West Nile virus. Viruses.

[103] Cook S., Bennett S.N., Holmes E.C., Chesse R., Moureau G., Lamballeria X. (2006). Isolation of a new strain of the flavivirus cell fusing agent virus in a natural mosquito population from Puerto Rico. J. Gen. Virol..

[104] Crabtree M.B., Sang R.C., Stollar V., Dunster L.M., Miller B.R. (2003). Genetic and phenotypic characterization of the newly described insect flavivirus, Kamiti River virus. Arch. Virol..

[105] Hoshino K., Isawa H., Tsuda Y., Yano K., Sasaki T., Yuda M., Takasaki T., Kobayashi M., Sawabe K. (2007). Genetic characterization of a new insect flavivirus isolated from *Culex pipiens* mosquito in Japan. Virology.

[106] Huhtamo E., Putkuri N., Kurkela S., Manni T., Vaheri A., Vapalahti O., Uzcategui N.Y. (2009). Characterization of a novel flavivirus from mosquitoes in northern europe that is related to mosquito-borne flaviviruses of the tropics. J. Virol..

[107] Bolling B.G., Eisen L., Moore C.G., Blair C.D. (2011). Insect-specific flaviviruses from *Culex mosquitoes* in Colorado, with evidence of vertical transmission. Am. J. Trop. Med. Hyg..

[108] Hobson-Peters J., Yam A.W., Lu J.W., Setoh Y.X., May F.J., Kurucz N., Walsh S., Prow N.A., Davis S.S., Weir R. (2013). A new insect-specific flavivirus from northern Australia suppresses replication of West Nile virus and Murray Valley encephalitis virus in co-infected mosquito cells. PLoS One.

[109] Calzolari M., Bonilauri P., Bellini R., Albieri A., Defilippo F., Maioli G., Galletti G., Gelati A., Barbieri I., Tamba M. (2010). Evidence of simultaneous circulation of West Nile and Usutu viruses in mosquitoes sampled in Emilia-Romagna region (Italy) in 2009. PLoS One.

[110] Newman C.M., Cerutti F., Anderson T.K., Hamer G.L., Walker E.D., Kitron U.D., Ruiz M.O., Brawn J.D., Goldberg T.L. (2011). *Culex flavivirus* and West Nile virus mosquito coinfection and positive ecological association in Chicago, United States. Vector Borne Zoonotic Dis..

[111] Bolling B.G., Olea-Popelka F.J., Eisen L., Moore C.G., Blair C.D. (2012). Transmission dynamics of an insect-specific flavivirus in a naturally infected *Culex pipiens* laboratory colony and effects of co-infection on vector competence for West Nile virus. Virology.

[112] Kent R.J., Crabtree M.B., Miller B.R. (2010). Transmission of West Nile virus by *Culex quinquefasciatus* say infected with *Culex Flavivirus* Izabal. PLoS Negl. Trop. Dis..

[113] Zou G., Zhang B., Lim P.Y., Yuan Z., Bernard K.A., Shi P.Y. (2009). Exclusion of West Nile virus superinfection through RNA replication. J. Virol..

[114] Weiss B., Aksoy S. (2011). Microbiome influences on insect host vector competence. Trends Parasitol..

[115] Hoffmann A.A., Montgomery B.L., Popovici J., Iturbe-Ormaetxe I., Johnson P.H., Muzzi F., Greenfield M., Durkan M., Leong Y.S., Dong Y. (2011). Successful establishment of Wolbachia in *Aedes populations* to suppress dengue transmission. Nature.

[116] Pan X., Zhou G., Wu J., Bian G., Lu P., Raikhel A.S., Xi Z. (2012). Wolbachia induces reactive oxygen species (ROS)-dependent activation of the Toll pathway to control dengue virus in the mosquito Aedes aegypti. Proc. Natl. Acad. Sci. USA.

[117] Glaser R.L., Meola M.A. (2010). The native Wolbachia endosymbionts of *Drosophila melanogaster* and *Culex quinquefasciatus* increase host resistance to West Nile virus infection. PLoS One.

[118] Hussain M., Lu G., Torres S., Edmonds J.H., Kay B.H., Khromykh A.A., Asgari S. (2013). Effect of wolbachia on replication of west nile virus in a mosquito cell line and adult mosquitoes. J. Virol..

[119] Bian G., Zhou G., Lu P., Xi Z. (2013). Replacing a native Wolbachia with a novel strain results in an increase in endosymbiont load and resistance to dengue virus in a mosquito vector. PLoS Negl. Trop. Dis..

[120] Pidiyar V.J., Jangid K., Patole M.S., Shouche Y.S. (2004). Studies on cultured and uncultured microbiota of wild culex quinquefasciatus mosquito midgut based on 16s ribosomal RNA gene analysis. Am. J. Trop. Med. Hyg..

[121] Ramirez J.L., Souza-Neto J., Torres C.R., Rovira J., Ortiz A., Pascale J.M., Dimopoulos G. (2012). Reciprocal tripartite interactions between the *Aedes aegypti* midgut microbiota, innate immune system and dengue virus influences vector competence. PLoS Negl. Trop. Dis..

[122] Ricci I., Damiani C., Capone A., DeFreece C., Rossi P., Favia G. (2012). Mosquito/microbiota interactions: From complex relationships to biotechnological perspectives. Curr. Opin. Microbiol..

[123] Ignatova E.A., Nagornaia S.S., Povazhnaia T.N., Ianishevskaia G.S. (1996). The yeast flora of blood-sucking mosquitoes. Mikrobiol. Z..

[124] Ricci I., Mosca M., Valzano M., Damiani C., Scuppa P., Rossi P., Crotti E., Cappelli A., Ulissi U., Capone A. (2011). Different mosquito species host *Wickerhamomyces anomalus* (Pichia anomala): Perspectives on vector-borne diseases symbiotic control. Antonie Van Leeuwenhoek.

[125] Garza-Hernandez J.A., Rodriguez-Perez M.A., Salazar M.I., Russell T.L., Adeleke M.A., de Luna-Santillana E.J., Reyes-Villanueva F. (2013). Vectorial capacity of *Aedes aegypti* for dengue virus type 2 is reduced with co-infection of *Metarhizium anisopliae*. PLoS Negl. Trop. Dis..

[126] Komar N., Dohm D.J., Turell M.J., Spielman A. (1999). Eastern equine encephalitis virus in birds: Relative competence of European starlings (*Sturnus vulgaris*). Am. J. Trop. Med. Hyg..

[127] Komar N., Langevin S., Hinten S., Nemeth N., Edwards E., Hettler D., Davis B., Bowen R., Bunning M. (2003). Experimental infection of North American birds with the New York 1999 strain of West Nile virus. Emerg. Infect. Dis..

[128] Dusek R.J., McLean R.G., Kramer L.D., Ubico S.R., Dupuis A.P., Ebel G.D., Guptill S.C. (2009). Prevalence of West Nile virus in migratory birds during spring and fall migration. Am. J. Trop. Med. Hyg..

[129] Kramer L.D., Bernard K.A. (2001). West Nile virus in the western hemisphere. Curr. Opin. Infect. Dis..

[130] Bernard K.A., Kramer L.D. (2001). West Nile virus activity in the United States, 2001. Viral Immunol..

[131] Ladeau S.L., Kilpatrick A.M., Marra P.P. (2007). West Nile virus emergence and large-scale declines of North American bird populations. Nature.

[132] Centers for Disease Control and Prevention Species of dead birds in which West Nile virus has been detected, United States, 1999–2012. http://www.cdc.gov/westnile/resources/pdfs/Bird%20Species%201999-2012.pdf.

[133] Reed L.M., Johansson M.A., Panella N., McLean R., Creekmore T., Puelle R., Komar N. (2009). Declining mortality in American crow (*Corvus brachyrhynchos*) following natural West Nile virus infection. Avian Dis..

[134] Komar N., Panella N.A., Burns J.E., Dusza S.W., Mascarenhas T.M., Talbot T.O. (2001). Serologic evidence for West Nile virus infection in birds in the New York City vicinity during an outbreak in 1999. Emerg. Infect. Dis..

[135] Malkinson M., Banet C., Weisman Y., Pokamunski S., King R., Drouet M.T., Deubel V. (2002). Introduction of West Nile virus in the Middle East by migrating white storks. Emerg. Infect. Dis..

[136] Tempelis C.H., Reeves W.C., Bellamy R.E., Lofy M.F. (1965). A three-year study of the feeding habits of *Culex tarsalis* in Kern County, California. Am. J. Trop. Med. Hyg..

[137] Tempelis C.H., Francy D.B., Hayes R.O., Lofy M.F. (1967). Variations in feeding patterns of seven culicine mosquitoes on vertebrate hosts in Weld and Larimer counties, Colorado. Am. J. Trop. Med. Hyg..

[138] Root J.J., Oesterle P.T., Nemeth N.M., Klenk K., Gould D.H., McLean R.G., Clark L., Hall J.S. (2006). Experimental infection of fox squirrels (*Sciurus niger*) with West Nile virus. Am. J. Trop. Med. Hyg..

[139] Gomez A., Kramer L.D., Dupuis A.P., Kilpatrick A.M., Davis L.J., Jones M.J., Daszak P., Aguirre A.A. (2008). Experimental infection of eastern gray squirrels (*Sciurus carolinensis*) with West Nile virus. Am. J. Trop. Med. Hyg..

[140] Gomez A., Kilpatrick A.M., Kramer L.D., Dupuis A.P., Maffei J.G., Goetz S.J., Marra P.P., Daszak P., Aguirre A.A. (2008). Land use and west nile virus seroprevalence in wild mammals. Emerg. Infect. Dis..

[141] Root J.J. (2013). West Nile virus associations in wild mammals: A synthesis. Arch. Virol..

[142] Smithburn K.C., Hughes T.P., Burke A.W., Paul J.H. (1940). A neurotropic virus isolated from the blood of a native of Uganda. Am. J. Trop. Med. Hyg..

[143] May F.J., Davis C.T., Tesh R.B., Barrett A.D. (2011). Phylogeography of West Nile virus: From the cradle of evolution in Africa to Eurasia, Australia, and the Americas. J. Virol..

[144] Papa A., Bakonyi T., Xanthopoulou K., Vazquez A., Tenorio A., Nowotny N. (2011). Genetic characterization of West Nile virus lineage 2, Greece, 2010. Emerg. Infect. Dis..

[145] Ciccozzi M., Peletto S., Cella E., Giovanetti M., Lai A., Gabanelli E., Acutis P.L., Modesto P., Rezza G., Platonov A.E. (2013). Epidemiological history and phylogeography of West Nile virus lineage 2. Infect. Genet. Evol..

[146] Beasley D.W., Li L., Suderman M.T., Barrett A.D. (2002). Mouse neuroinvasive phenotype of West Nile virus strains varies depending upon virus genotype. Virology.

[147] Bakonyi T., Hubalek Z., Rudolf I., Nowotny N. (2005). Novel flavivirus or new lineage of West Nile virus, central Europe. Emerg. Infect. Dis..

[148] Hubalek Z., Rudolf I., Bakonyi T., Kazdova K., Halouzka J., Sebesta O., Sikutova S., Juricova Z., Nowotny N. (2010). Mosquito (Diptera: Culicidae) surveillance for arboviruses in an area endemic for West Nile (Lineage Rabensburg) and Tahyna viruses in Central Europe. J. Med. Entomol..

[149] Aliota M.T., Jones S.A., Dupuis A.P., Ciota A.T., Hubalek Z., Kramer L.D. (2012). Characterization of Rabensburg virus, a flavivirus closely related to West Nile virus of the Japanese encephalitis antigenic group. PLoS One.

[150] Prilipov A.G., Kinney R.M., Samokhvalov E.I., Savage H.M., Al’khovskii S.V., Tsuchiya K.R., Gromashevskii V.L., Sadykova G.K., Shatalov A.G., Vyshemirskii O.I. (2002). Analysis of new variants of West Nile fever virus. Vopr. Virusol..

[151] Vazquez A., Sanchez-Seco M.P., Ruiz S., Molero F., Hernandez L., Moreno J., Magallanes A., Tejedor C.G., Tenorio A. (2010). Putative new lineage of west nile virus, Spain. Emerg. Infect. Dis..

[152] Bondre V.P., Jadi R.S., Mishra A.C., Yergolkar P.N., Arankalle V.A. (2007). West Nile virus isolates from India: Evidence for a distinct genetic lineage. J. Gen. Virol..

[153] Scherret J.H., Poidinger M., Mackenzie J.S., Broom A.K., Deubel V., Lipkin W.I., Briese T., Gould E.A., Hall R.A. (2001). The relationships between West Nile and Kunjin viruses. Emerg. Infect. Dis..

[154] Charrel R.N., Brault A.C., Gallian P., Lemasson J.J., Murgue B., Murri S., Pastorino B., Zeller H., de Chesse R., de Micco P. (2003). Evolutionary relationship between Old World West Nile virus strains. Evidence for viral gene flow between Africa, the Middle East, and Europe. Virology.

[155] Malkinson M., Banet C., Weisman Y., Pokamonski S., King R., Deubel V. (2001). Intercontinental transmission of West Nile virus by migrating white storks. Emerg. Infect. Dis..

[156] McMullen A.R., May F.J., Li L., Guzman H., Bueno R., Dennett J.A., Tesh R.B., Barrett A.D. (2011). Evolution of new genotype of West Nile virus in North America. Emerg. Infect. Dis..

[157] Armstrong P.M., Vossbrinck C.R., Andreadis T.G., Anderson J.F., Pesko K.N., Newman R.M., Lennon N.J., Birren B.W., Ebel G.D., Henn M.R. (2011). Molecular evolution of West Nile virus in a northern temperate region: Connecticut, USA 1999–2008. Virology.

[158] Brault A.C., Huang C.Y., Langevin S.A., Kinney R.M., Bowen R.A., Ramey W.N., Panella N.A., Holmes E.C., Powers A.M., Miller B.R. (2007). A single positively selected West Nile viral mutation confers increased virogenesis in American crows. Nat. Genet..

[159] Pesko K.N., Ebel G.D. (2012). West Nile virus population genetics and evolution. Infect. Genet. Evol..

[160] Scholle F., Girard Y.A., Zhao Q.Z., Higgs S., Mason P.W. (2004). *trans*-Packaged West Nile virus-like particles: Infectious properties in vitro and in infected mosquito vectors. J. Virol..

[161] Ciota A.T., Ehrbar D.J., van Slyke G.A., Payne A.F., Willsey G.G., Viscio R.E., Kramer L.D. (2012). Quantification of intrahost bottlenecks of West Nile virus in Culex pipiens mosquitoes using an artificial mutant swarm. Infect. Genet. Evol..

[162] Brackney D.E., Pesko K.N., Brown I.K., Deardorff E.R., Kawatachi J., Ebel G.D. (2011). West Nile virus genetic diversity is maintained during transmission by Culex pipiens quinquefasciatus Mosquitoes. PLoS One.

[163] Drake J.W. (1993). Rates of Spontaneous Mutation Among RNA Viruses. Proc. Natl. Acad. Sci. USA.

[164] Jerzak G.V., Bernard K., Kramer L.D., Shi P.Y., Ebel G.D. (2007). The West Nile virus mutant spectrum is host-dependant and a determinant of mortality in mice. Virology.

[165] Bertolotti L., Kitron U., Goldberg T.L. (2007). Diversity and evolution of West Nile virus in Illinois and the United States, 2002–2005. Virology.

[166] Amore G., Bertolotti L., Hamer G.L., Kitron U.D., Walker E.D., Ruiz M.O., Brawn J.D., Goldberg T.L. (2010). Multi-year evolutionary dynamics of West Nile virus in suburban Chicago, USA, 2005–2007. Philos. Trans. R. Soc. Lond. B Biol. Sci..

[167] Jerzak G., Bernard K.A., Kramer L.D., Ebel G.D. (2005). Genetic variation in West Nile virus from naturally infected mosquitoes and birds suggests quasispecies structure and strong purifying selection. J. Gen. Virol..

[168] Ciota A.T., Jia Y., Payne A.F., Jerzak G., Davis L.J., Young D.S., Ehrbar D., Kramer L.D. (2009). Experimental passage of St. Louis encephalitis virus *in vivo* in mosquitoes and chickens reveals evolutionarily significant virus characteristics. PLoS One.

[169] Ciota A.T., Ehrbar D.J., van Slyke G.A., Willsey G.G., Kramer L.D. (2012). Cooperative interactions in the West Nile virus mutant swarm. BMC Evol. Biol..

[170] Ciota A.T., Lovelace A.O., Jones S.A., Payne A., Kramer L.D. (2007). Adaptation of two flaviviruses results in differences in genetic heterogeneity and virus adaptability. J. Gen. Virol..

[171] Brackney D.E., Beane J.E., Ebel G.D. (2009). RNAi targeting of West Nile virus in mosquito midguts promotes virus diversification. PLoS Pathog..

[172] Fitzpatrick K.A., Deardorff E.R., Pesko K., Brackney D.E., Zhang B., Bedrick E., Shi P.Y., Ebel G.D. (2010). Population variation of West Nile virus confers a host-specific fitness benefit in mosquitoes. Virology.

[173] Ciota A.T., Lovelace A.O., Jia Y., Davis L.J., Young D.S., Kramer L.D. (2008). Characterization of mosquito-adapted West Nile virus. J. Gen. Virol..

[174] Bugbee L.M., Forte L.R. (2004). The discovery of West Nile virus in overwintering *Culex pipiens* (Diptera: Culicidae) mosquitoes in Lehigh County, Pennsylvania. J. Am. Mosq. Control Assoc..

[175] Farajollahi A., Crans W.J., Nickerson D., Bryant P., Wolf B., Glaser A., Andreadis T.G. (2005). Detection of West Nile virus RNA from the louse fly Icosta Americana (Diptera: Hippoboscidae). J. Am. Mosq. Control Assoc..

[176] Reisen W.K., Fang Y., Lothrop H.D., Martinez V.M., Wilson J., O’Connor P., Carney R., Cahoon-Young B., Shafii M., Brault A.C. (2006). Overwintering of West Nile Virus in Southern California. J. Med. Entomol..

[177] Dohm D.J., Sardelis M.R., Turell M.J. (2002). Experimental vertical transmission of West Nile virus by *Culex pipiens* (Diptera: Culicidae). J. Med. Entomol..

[178] Nelms B.M., Fechter-Leggett E., Carroll B.D., Macedo P., Kluh S., Reisen W.K. (2013). Experimental and natural vertical transmission of West Nile virus by California Culex (Diptera: Culicidae) mosquitoes. J. Med. Entomol..

[179] Ciota A.T., Koch E.M., Willsey G.G., Davis L.J., Jerzak G.V., Ehrbar D.J., Wilke C.O., Kramer L.D. (2011). Temporal and spatial alterations in mutant swarm size of St. Louis encephalitis virus in mosquito hosts. Infect. Genet. Evol..

[180] Davis C.T., Ebel G.D., Lanciotti R.S., Brault A.C., Guzman H., Siirin M., Lambert A., Parsons R.E., Beasley D.W., Novak R.J. (2005). Phylogenetic analysis of North American West Nile virus isolates, 2001–2004: Evidence for the emergence of a dominant genotype. Virology.

[181] Twiddy S.S., Pybus O.G., Holmes E.C. (2003). Comparative population dynamics of mosquito-borne flaviviruses. Infect. Genet. Evol..

[182] Woolhouse M.E., Taylor L.H., Haydon D.T. (2001). Population biology of multihost pathogens. Science.

[183] Scott T.W., Weaver S.C., Mallampalli V.L., Morse S.S. (1994). Evolution of Mosquito-Borne Viruses. The Evolutionary Biology of Viruses.

[184] Levins R. (1968). Evolution in Changing Environments.

[185] Ciota A.T., Lovelace A.O., Ngo K.A., Le A.N., Maffei J.G., Franke M.A., Payne A.F., Jones S.A., Kauffman E.B., Kramer L.D. (2007). Cell-specific adaptation of two flaviviruses following serial passage in mosquito cell culture. Virology.

[186] Deardorff E.R., Fitzpatrick K.A., Jerzak G.V., Shi P.Y., Kramer L.D., Ebel G.D. (2011). West Nile virus experimental evolution *in vivo* and the trade-off hypothesis. PLoS Pathog..

[187] Wilke C.O., Wang J.L., Ofria C., Lenski R.E., Adami C. (2001). Evolution of digital organisms at high mutation rates leads to survival of the flattest. Nature.

[188] Tsetsarkin K.A., McGee C.E., Volk S.M., Vanlandingham D.L., Weaver S.C., Higgs S. (2009). Epistatic roles of E2 glycoprotein mutations in adaption of chikungunya virus to *Aedes albopictus* and Ae. *Aegypti mosquitoes*. PLoS One.

[189] Weaver S.C., Reisen W.K. (2010). Present and future arboviral threats. Antivir. Res..

[190] Aaskov J., Buzacott K., Thu H.M., Lowry K., Holmes E.C. (2006). Long-term transmission of defective RNA viruses in humans and Aedes mosquitoes. Science.

[191] Lambrechts L. (2010). Dissecting the genetic architecture of host-pathogen specificity. PLoS Pathog..

[192] Lambrechts L., Quillery E., Noel V., Richardson J.H., Jarman R.G., Scott T.W., Chevillon C. (2013). Specificity of resistance to dengue virus isolates is associated with genotypes of the mosquito antiviral gene Dicer-2. Proc. Biol. Sci..

[193] Snappin K.W., Holmes E.C., Young D.S., Bernard K.A., Kramer L.D., Ebel G.D. (2007). Declining growth rate of West Nile virus in North America. J. Virol..

[194] Centers for Disease Control and Prevention West Nile virus disease cases and deaths reported to CDC by year and clinical presentation, 1999–2012. http://www.cdc.gov/westnile/resources/pdfs/cummulative/99_2012_CasesAndDeathsClinicalPresentationHumanCases.pdf.

[195] Duggal N.K., D’Anton M., Xiang J., Seiferth R., Day J., Nasci R., Brault A.C. (2013). Sequence analyses of 2012 west nile virus isolates from Texas fail to associate viral genetic factors with outbreak magnitude. Am. J. Trop. Med. Hyg..

